# A Mathematical Model of Cysteine-Driven Metabolic Adaptation to Hypoxia in Ovarian Cancer

**DOI:** 10.3390/bioengineering13030300

**Published:** 2026-03-04

**Authors:** José A. Rodrigues, Sofia C. Nunes, Cristiano Ramos, Luis G. Gonçalves, Jacinta Serpa

**Affiliations:** 1 CIMA and Department of Mathematics of Instituto Superior de Engenharia de Lisboa–ISEL, Polytechnic University of Lisbon, Rua Conselheiro Emídio Navarro, 1, 1959-007 Lisbon, Portugal; 2 CIMOSM, ISEL—Centro de Investigação em Modelação e Optimização de Sistemas Multifuncionais, Instituto Superior de Engenharia de Lisboa, 1959-007 Lisboa, Portugal; 3 NOVA Medical School|Faculdade de Ciências Médicas, Universidade NOVA de Lisboa, Campo dos Mártires da Pátria 130, 1169-056 Lisbon, Portugal; 4 Instituto Português de Oncologia de Lisboa Francisco Gentil (IPOLFG), Rua Prof. Lima Basto, 1099-023 Lisbon, Portugal; 5 Instituto de Tecnologia Química e Biológica António Xavier (ITQB NOVA), Avenida da República (EAN), 2780-157 Oeiras, Portugal

**Keywords:** mathematical modelling, nonlinear dynamics, model calibration, systems biology modelling, quantitative redox modelling, metabolic network dynamics, cysteine metabolism, hypoxia, metabolic adaptation, redox homeostasis, ovarian cancer

## Abstract

Ovarian cancer progression is strongly influenced by tumour hypoxia and associated oxidative stress. Experimental evidence indicates that cysteine availability supports ovarian cancer cell fitness under hypoxic conditions, yet the quantitative integration of cysteine metabolism, redox control, and energetic maintenance remains incompletely understood. We present a reduced mechanistic mathematical model describing intracellular cysteine allocation between glutathione (GSH) synthesis and hydrogen sulfide production under experimentally imposed hypoxia. The model integrates extracellular cysteine uptake, GSH-dependent reactive oxygen species (ROS) detoxification, hypoxia-amplified ROS generation, and redox-modulated ATP maintenance. Parameter estimation was performed using experimentally derived extracellular metabolite fluxes measured over a 24 h interval. Uncertainty was assessed via bootstrap resampling, and variance-based sensitivity analysis was conducted within (patho)physiologically constrained parameter domains. The calibrated model reproduces extracellular fluxes with relative deviations below 7% and identifies GSH synthesis capacity as the dominant determinant of ATP maintenance within experimentally supported ranges. Hydrogen sulfide (H_2_S) production exerts a secondary stabilising influence, whereas hypoxia-driven ROS amplification negatively impacts energetic state. Numerical continuation across hypoxia levels reveals distinct qualitative response regions but does not imply a formal bifurcation structure. Importantly, intracellular metabolite dynamics are inferred as latent variables consistent with extracellular constraints and established biochemical knowledge; the model does not uniquely identify intracellular pool sizes or enzyme kinetics. The framework therefore provides flux-consistent mechanistic plausibility rather than direct intracellular validation. This systems-level analysis supports cysteine allocation as a quantitatively influential control point in hypoxic adaptation and establishes a constrained modelling framework for subsequent metabolic network expansions and experimental validation.

## 1. Introduction

Ovarian cancer remains one of the deadliest gynaecological malignancies, with survival rates that have improved only modestly over recent decades [1]. A hallmark of advanced ovarian tumours is the presence of hypoxic regions, arising from abnormal vasculature and rapid tumour growth. Hypoxia is a potent driver of tumour progression, metabolic reprogramming, and resistance to therapy [2,3].

One major consequence of hypoxia is increased oxidative stress, resulting from impaired mitochondrial electron transport and altered redox homeostasis. To survive under such conditions, cancer cells activate adaptive metabolic programmes that enhance antioxidant capacity and sustain energy production [4].

Cysteine plays a central role in these adaptations. It is the rate-limiting precursor for glutathione (GSH), the major intracellular antioxidant, and a substrate for hydrogen sulfide (H_2_S) production, which has been implicated in redox signalling and mitochondrial bioenergetics [5]. Experimental studies have demonstrated that ovarian cancer cells increase their dependence on cysteine under hypoxia, promoting survival and resistance to platinum-based chemotherapy [6,7].

Despite these insights, a quantitative understanding of how cysteine-dependent metabolic pathways interact to determine cellular fitness under hypoxia is still lacking. Mathematical modelling provides a powerful approach to integrate experimental data, formalise mechanistic hypotheses, and identify key metabolic control points.

Here, we develop a mechanistic mathematical model describing cysteine-driven metabolic adaptation to hypoxia in ovarian cancer cells. The model is calibrated using experimental metabolomic data and analysed through numerical simulation and global sensitivity analysis.

## 2. Experimental Data and Biological Basis

The model is informed by the levels of extracellular metabolites obtained from the exometabolome of ovarian cancer cell lines (ES2; CRL-1978, and OVCAR-3; HTB-161 from American Type Culture Collection-ATCC) cultured under normoxic and hypoxic conditions, with and without cysteine supplementation. The culture conditions and the experiments needed to obtain the exometabolome profile are already published [7]. Metabolite concentrations were measured in the culture medium at defined time points, including amino acids, such as cysteine, methionine, glutamate, glutamine, and glucose.

Changes in extracellular concentrations over a 24 h period were used to infer apparent uptake and secretion fluxes according to

(1) $$v_{i}^{\mathrm{app}}=\frac{M_{i}\left(t_{0}\right)-M_{i}\left(t_{f}\right)}{\Delta t},$$

where $M_{i}$ denotes the extracellular concentration of metabolite *i*.

The experimental dataset used in this study consists of extracellular metabolite concentrations measured at the beginning (0 h) and end (24 h) of the culture period under defined experimental conditions, including hypoxia and cysteine supplementation. These data provide quantitative information on the net metabolite uptake and secretion rates over the experimental time window.

For each measured extracellular metabolite *i*, an apparent mean flux was computed as

(2) $$v_{i}^{\exp}=\frac{M_{i}\left(0\right)-M_{i}\left(24\right)}{24},$$

where concentrations are expressed in mM and time in hours. These apparent fluxes constitute the primary experimental observables used for quantitative calibration of the mathematical model.

Intracellular metabolite concentrations are not directly measured and are therefore treated as latent variables, inferred indirectly through consistency between the model-predicted fluxes and experimentally observed extracellular fluxes.

These data indicate enhanced cysteine consumption under hypoxia, accompanied by coordinated changes in redox-related metabolites, supporting the central role of cysteine in hypoxic adaptation.

## 3. Mathematical Model Formulation

### 3.1. State Variables

The model describes the temporal evolution of five intracellular variables:

- C(t): intracellular cysteine concentration,
- G(t): reduced glutathione (GSH),
- R(t): reactive oxygen species (ROS),
- S(t): hydrogen sulfide (H_2_S),
- A(t): ATP concentration (proxy for cellular energetic state).

Hypoxia is represented by a dimensionless parameter H∈[0,1], corresponding to normoxia and hypoxia, respectively. In this first modelling framework, hypoxia is treated as a fixed external condition, reflecting experimentally imposed oxygen levels rather than dynamically evolving oxygen availability. While oxygen concentration may vary in space and time in vivo, explicitly modelling oxygen transport and consumption is beyond the scope of the present work and will be addressed in future extensions of the model.

### 3.2. Model Equations

The system is formulated as a set of ordinary differential equations:

Cysteine dynamics

(3) $$\frac{dC}{dt}=k_{\mathrm{in}}C_{\mathrm{ext}}-\frac{V_{G}C}{K_{G}+C}-\frac{V_{S}C}{K_{S}+C}-k_{C}C,$$

where cysteine is taken up from the extracellular medium and consumed by glutathione and H_2_S synthesis, as well as basal metabolic processes.

Glutathione dynamics

(4) $$\frac{dG}{dt}=\left(1+\alpha H\right)\frac{V_{G}C}{K_{G}+C}-k_{R}GR$$

Hypoxia enhances the flux of cysteine toward GSH synthesis, reflecting increased antioxidant demand.

ROS dynamics

(5) $$\frac{dR}{dt}=\left(1+\beta H\right)k_{ROS}-k_{R}GR$$

The term $k_{R}GR$ represents mass-action GSH-dependent ROS neutralisation, where the detoxification rate scales proportionally with both reduced glutathione concentration and ROS abundance. The explicitly multiplicative structure removes notational ambiguity and ensures direct correspondence between model equations and biochemical interaction terms.

Hydrogen sulfide dynamics

(6) $$\frac{dS}{dt}=\frac{V_{S}C}{K_{S}+C}-k_{S}S.$$

ATP dynamics

(7) $$\frac{dA}{dt}=k_{A,O}\;G\left(1-H\right)+k_{A,S}\;S-k_{A,d}\;A$$

ATP production is not assumed to be directly generated by GSH. Instead, GSH is modelled as a permissive regulator of mitochondrial function through its role in redox buffering. The term $k_{A,O}G\left(1-H\right)$ captures the experimentally supported requirement of adequate redox control for efficient oxidative phosphorylation. Thus, *G* acts as a modulatory factor rather than as a direct energetic substrate.

This formulation separates oxygen-dependent ATP production from hypoxia-tolerant H_2_S-supported energetic pathways, reflecting experimental evidence that H_2_S can contribute to mitochondrial electron transport even under limited oxygen availability.

It should be noted that the structure of the glutathione equation favours stability of GSH levels provided cysteine is available, reflecting a strong homeostatic control. The robustness of this behaviour is further explored numerically in Section 6.

The system of Equations (3)–(7) defines a reduced intracellular metabolic network in which cysteine serves as a branching substrate between two functionally distinct pathways: redox buffering through GSH synthesis and alternative mitochondrial support through H_2_S production. Hypoxia is incorporated as an external parametric driver that increases ROS generation and redox demand while simultaneously impairing oxygen-dependent ATP production.

**Figure 1:**
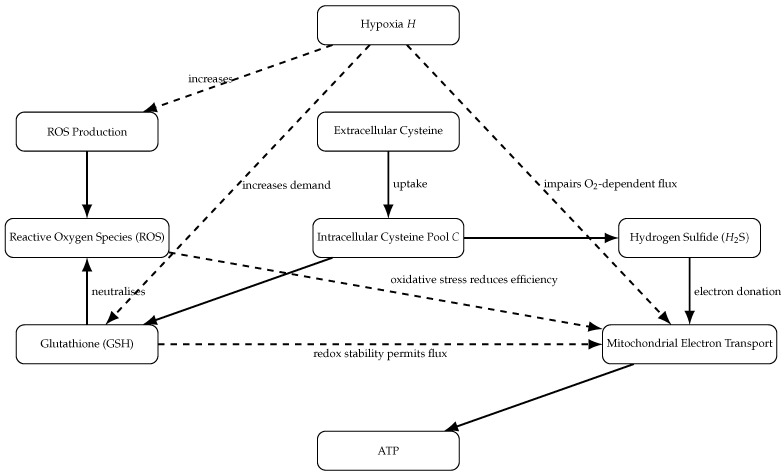
Schematic representation of the reduced cysteine-centred metabolic network. Solid arrows denote material fluxes. Dashed arrows denote regulatory modulation. Extracellular cysteine is imported into the intracellular pool and partitioned between glutathione (GSH) synthesis and hydrogen sulfide (H_2_S) production. GSH participates in ROS detoxification, contributing indirectly to ATP maintenance via preservation of redox stability. H_2_S supports ATP production through mitochondrial electron transport. Hypoxia (*H*) increases ROS production and amplifies demand for GSH-mediated redox buffering.

The structural organisation of this reduced network is summarised in Figure 1. The schematic provides a graphical correspondence between the biochemical processes and the mathematical terms appearing in the differential equations. In particular, it visualises cysteine allocation, GSH-dependent ROS neutralisation, H_2_S-supported mitochondrial flux, and hypoxia-mediated modulation. No additional pathways are introduced beyond those explicitly represented in Equations (3)–(7); the diagram therefore functions as a structural abstraction of the model rather than as an expanded biological map.

All state variables (*C*, *G*, *R*, *S*, *A*) are expressed in mM. Time is expressed in *h*. Uptake and first-order turnover parameters ($k_{in}$, $k_{C}$, $k_{S}$, $k_{A,d}$) have units of h^−1^ when multiplied by concentrations. Maximal flux parameters ($V_{G}$, $V_{S}$) have units of mM h^−1^, and Michaelis constants ($K_{G}$, $K_{S}$) have units of mM. Each term in Equations (3)–(7) therefore has units of mM h^−1^, ensuring dimensional consistency throughout the dynamical system.

The dimensional homogeneity of each equation was independently verified by expressing all flux terms in units of mM h^−1^. No composite term combines parameters and variables with inconsistent units.

## 4. Parameter Estimation and Numerical Methods

### 4.1. Selection of Parameters for Calibration

Parameter estimation was restricted to a subset of model parameters that are directly informed by the available experimental data. Specifically, the following parameters were estimated from the data: the cysteine uptake rate $k_{\mathrm{in}}$, the maximal flux of cysteine toward glutathione synthesis $V_{G}$, the maximal flux of cysteine toward hydrogen sulfide production $V_{S}$, the basal rate of reactive oxygen species generation $k_{\mathrm{ROS}}$, and the hypoxia-induced amplification factor β.

All remaining parameters were fixed to biologically plausible values obtained from the literature. This parameter selection strategy reduces model dimensionality, improves numerical identifiability, and ensures that estimated parameters are constrained by experimental observations.

### 4.2. Definition of Experimental Observables

The experimental observables used for model calibration are the apparent extracellular uptake and secretion fluxes computed from concentration changes over a 24 h period. For each experimental condition, model simulations were performed over the same time interval, and the mean model-predicted fluxes were computed as

(8) $${\bar{v}}_{i}^{\mathrm{model}}=\frac{1}{24}\int_{0}^{24}v_{i}\left(t\right)\;dt.$$

These quantities were directly compared with the experimentally inferred fluxes $v_{i}^{\exp}$, ensuring consistency between the numerical model and the experimental dataset.

### 4.3. Objective Function for Parameter Estimation

Parameter estimation was formulated as a constrained optimisation problem, in which the model parameter vector θ was determined by minimising an objective function that quantified the mismatch between experimentally inferred extracellular metabolite fluxes and those predicted by the mathematical model.

For each extracellular metabolite i∈M and each experimental condition k∈C, the experimental observable is the apparent mean flux $v_{i,k}^{\exp}$, computed from extracellular concentration changes over a 24 h interval. Given a parameter set θ, the model was numerically integrated over the same time window, and the corresponding mean model-predicted flux was computed as

(9) $${\bar{v}}_{i,k}^{\mathrm{model}}\left(\theta \right)=\frac{1}{24}\int_{0}^{24}v_{i,k}\left(t;\theta \right)\;dt.$$

The objective function was defined as

(10) $$J\left(\theta \right)=\sum_{i\in M}\sum_{k\in C}{\left(\frac{{\bar{v}}_{i,k}^{\mathrm{model}}\left(\theta \right)-v_{i,k}^{\exp}}{v_{i,k}^{\exp}}\right)}^{2},$$

and parameter estimation was performed by minimising J(θ) over a biologically admissible parameter domain.

The use of relative squared errors ensures that all metabolites contribute comparably to the objective function, independently of their absolute concentration levels or units, thereby preventing dominant contributions from high-abundance metabolites. This formulation is particularly appropriate for extracellular metabolomic data, where concentration ranges may differ by several orders of magnitude across metabolites.

By calibrating the model to experimentally inferred fluxes rather than intracellular concentrations, the optimisation problem remains fully consistent with the available data while allowing intracellular metabolite dynamics to be inferred implicitly. This flux-based calibration strategy reduces sensitivity to unknown intracellular pool sizes and measurement noise, and yields parameter estimates that are directly anchored to experimentally observable quantities.

Finally, the structure of the objective function allows for direct comparison across experimental conditions, including normoxia versus hypoxia and cysteine-supplemented versus control media, thereby ensuring that the estimated parameters capture the coordinated metabolic adaptations observed experimentally.

### 4.4. Numerical Optimisation and Uncertainty Analysis

Minimisation of the objective function defined in Section 4.3 was performed using nonlinear least-squares optimisation. To mitigate the risk of convergence to local minima, the optimisation procedure was repeated from multiple initial parameter guesses sampled uniformly within predefined biologically admissible ranges. These ranges were selected on the basis of biochemical plausibility and prior knowledge reported in the literature, particularly regarding hypoxia-induced metabolic regulation and cysteine-centred redox control [2,5].

Parameter bounds were chosen to ensure the positivity and physiological relevance of all estimated quantities, while remaining sufficiently wide to avoid artificial constraint of the optimisation landscape. This approach is particularly important in nonlinear metabolic models, where parameter interdependencies may give rise to flat or weakly identifiable directions in parameter space.

Uncertainty in the estimated parameter values was assessed using a bootstrap resampling strategy based on experimental flux variability. Specifically, experimental extracellular fluxes were resampled assuming independent perturbations consistent with the observed experimental variability, and the calibration procedure was repeated for each resampled dataset. The resulting ensemble of parameter estimates was used to compute confidence intervals and summary statistics for each calibrated parameter.

To further characterise the parameter dependencies, pairwise correlations between estimated parameters were computed from the bootstrap ensemble. This analysis was used to identify potential compensatory mechanisms within the model, in particular between the maximal flux of cysteine toward glutathione synthesis and the maximal flux toward hydrogen sulfide production. Such compensatory behaviour is biologically plausible, as both pathways draw from the same intracellular cysteine pool and have been implicated in hypoxia-associated redox and metabolic adaptation [5].

Together, this optimisation and uncertainty analysis framework ensures that the reported parameter estimates are not only consistent with experimental observations but also quantitatively robust and interpretable within the known biological constraints of hypoxia-driven metabolic reprogramming [2].

While the multi-start optimisation strategy ensures convergence towards a consistent minimising parameter vector, numerical agreement alone does not guarantee the practical recoverability of individual parameters. In nonlinear metabolic models constrained by limited observables, assessment of the parameter dispersion under experimental variability is therefore required to evaluate practical identifiability. This motivates the bootstrap-based analysis presented below.

### 4.5. Practical Identifiability Analysis

Because parameter estimation relies on extracellular flux measurements evaluated at two time points (0 h and 24 h), assessment of practical identifiability is essential. To quantify the estimation robustness, a nonparametric bootstrap procedure was implemented by resampling the experimentally inferred extracellular fluxes within their observed variability and recalibrating the model for each synthetic dataset.

Let ${\hat{\theta }}^{\left(b\right)}$ denote the parameter vector estimated from bootstrap replicate *b*, with b=1,…,B. In the present study, B=500 bootstrap replicates were generated. For each calibrated parameter $\theta_{i}$, empirical 95% confidence intervals were computed using the percentile method, yielding

$$\theta_{i}^{2.5\%},\;\theta_{i}^{97.5\%}.$$

The resulting bootstrap-based summary statistics are reported in Table 1. All calibrated parameters exhibit bounded and non-degenerate confidence intervals.

The coefficients of variation remain below 8% for all calibrated parameters, indicating practical identifiability within the flux-constrained reduced model. The moderate negative correlation between $k_{\mathrm{in}}$ and $V_{G}$ (ρ≈−0.67) reflects the partial compensatory allocation of intracellular cysteine flux. However, no near-singular parameter dependencies (defined as |ρ|>0.9) were detected, indicating the absence of severe compensation or practical non-identifiability within the biologically admissible parameter domain.

The hypoxia amplification parameter β exhibits particularly low dispersion across bootstrap replicates. This reflects the strong constraint imposed by the hypoxia-dependent ROS amplification term within the flux-calibrated objective function. The narrow interval does not arise from boundary saturation, as the optimisation bounds allow substantially wider admissible variation.

From a structural perspective, identifiability of the hypoxia amplification parameter β follows directly from the steady-state form of the ROS equation. At equilibrium,

(11) $$k_{R}GR=\left(1+\beta H\right)\;k_{\mathrm{ROS}},$$

so that, for H≠0,

(12) $$\beta =\frac{k_{R}GR}{Hk_{\mathrm{ROS}}}-\frac{1}{H}.$$

Hence, β is algebraically recoverable from the steady-state redox balance once $k_{\mathrm{ROS}}$ is independently estimated, and simulations include at least two distinct hypoxia levels (e.g., normoxia H=0 and hypoxia H>0). The narrow bootstrap interval therefore reflects the strong structural constraint imposed by the redox steady-state relation rather than optimisation artefacts or boundary saturation effects.

To further characterise the local curvature properties of the objective function near the optimum, the Hessian matrix of J(θ) was numerically approximated at the minimising parameter vector. The resulting condition number ($\kappa \approx 10^{2}$) indicates anisotropic but well-conditioned local curvature, with no evidence of flat directions compatible with practical ill-posedness in the calibrated parameter neighbourhood.

Taken together, these results demonstrate controlled parameter compensation without degeneracy of the inverse problem under the imposed cysteine-centred reduction architecture.

## 5. Sensitivity Analysis

Sensitivity analysis was conducted to quantify how parameter uncertainty propagates to model outputs associated with energetic and redox state. Variance-based Sobol indices were computed within physiologically admissible parameter domains. Sampling was performed using quasi-random Sobol sequences across bounded intervals reflecting both biochemical plausibility and bootstrap-derived uncertainty ranges. Unlike purely structural global sensitivity analysis over arbitrarily wide domains, the present approach quantifies variance propagation within experimentally constrained regions of parameter space. The resulting indices therefore represent biologically relevant control coefficients rather than unrestricted structural sensitivities.

### 5.1. Sensitivity Method

Global sensitivity analysis was performed using first-order Sobol indices to quantify the relative contribution of individual model parameters to the variance of selected model outputs. This variance-based approach is well suited for nonlinear dynamical systems, as it captures parameter influence over the full parameter range while accounting for non-additive effects.

Sensitivity analysis was carried out with respect to the subset of parameters calibrated against the experimental data, while all remaining parameters were fixed at their nominal values. Parameter sampling was performed around the calibrated parameter vector, with each parameter independently varied within a restricted interval of ±20–30% of its estimated value. These bounds were chosen to reflect the experimentally plausible variability, as inferred from the bootstrap-based uncertainty analysis.

For each sampled parameter set, the model was numerically integrated over a 24 h period under hypoxic conditions, and selected outputs were computed from the resulting trajectories. First-order Sobol indices were then computed for each parameter–output pair, measuring the fraction of the total output variance attributable to variation in that parameter alone.

By conditioning the sensitivity analysis on the calibrated parameter values and experimentally constrained parameter ranges, the resulting Sobol indices reflect physiologically relevant control mechanisms rather than purely structural properties of the model. This approach ensures that parameters identified as influential exert a quantitatively meaningful impact on model behaviour within the biological context considered.

To assess robustness beyond the physiologically constrained interval, an auxiliary wide-domain sensitivity scan was performed by varying the calibrated parameters within ±100% of their estimated values while preserving positivity. Although absolute variance magnitudes increased under this broader exploration, the ranking of first-order Sobol indices remained qualitatively preserved, with $V_{G}$ retaining dominant influence on ATP-related outputs. This indicates that the identified control hierarchy is not exclusively an artefact of narrow parameter bounds.

### 5.2. Sensitivity Outputs

Sensitivity was evaluated with respect to four complementary outputs:

(i) Final ATP level at 24 h:
  (13) $$A_{24}:=A\left(24\right).$$
(ii) Cumulative ATP availability:
  (14) $${\mathrm{ATP}}_{\mathrm{int}}=\int_{0}^{24}A\left(t\right)\;dt.$$
(iii) Cumulative ROS burden:
  (15) $${\mathrm{ROS}}_{\mathrm{int}}=\int_{0}^{24}R\left(t\right)\;dt.$$
(iv) Energetic–redox balance ratio:
  (16) $${\mathrm{Fitness}}_{\mathrm{ratio}}=\frac{{\mathrm{ATP}}_{\mathrm{int}}}{{\mathrm{ROS}}_{\mathrm{int}}}.$$

Explicitly,

(17) $${\mathrm{Fitness}}_{\mathrm{ratio}}=\frac{\int_{0}^{24}A\left(t\right)\;dt}{\int_{0}^{24}R\left(t\right)\;dt}.$$

This multidimensional output set distinguishes the parameters predominantly affecting energy maintenance from those modulating oxidative burden or energetic–redox trade-offs.

Across all outputs, $V_{G}$ consistently exhibits the largest first-order Sobol index within the explored admissible domain, followed by $V_{S}$. The hypoxia amplification parameter β contributes negatively to ATP-related outputs, reflecting its role in ROS escalation.

The dominance of glutathione synthesis therefore persists within experimentally constrained domains, although it should be interpreted as conditional upon the reduced cysteine-centred architecture and bounded parameter space.

## 6. Numerical Results

### 6.1. Model Calibration Against Experimental Extracellular Fluxes

The calibrated model reproduces the experimentally inferred extracellular uptake and secretion fluxes across all experimental conditions, with relative deviations below 7% for all measured metabolites (Table 2). Experimental fluxes ($v_{exp}$) were computed from extracellular metabolite concentration changes over a 24 h period, while the mean model-predicted fluxes (${\bar{v}}_{model}$) correspond to the time-averaged reaction rates obtained from numerical simulations of the model equations. The close agreement between $v_{exp}$ and ${\bar{v}}_{model}$, including cysteine uptake rates under hypoxia and cysteine supplementation, provides quantitative validation of the parameter estimation procedure and supports the use of the calibrated model for subsequent dynamical and sensitivity analyses.

### 6.2. Dynamic Behaviour of the Cysteine-Centred Metabolic Network Under Hypoxia

All dynamic simulations reported below were performed using parameters calibrated against experimental extracellular flux data.

**Figure 2:**
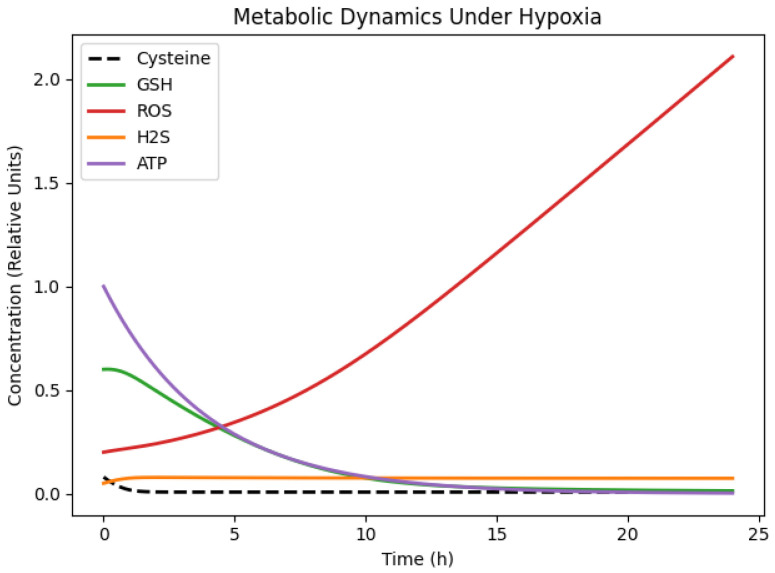
Simulated temporal dynamics of intracellular cysteine, glutathione (GSH), reactive oxygen species (ROS), hydrogen sulfide (H_2_S), and ATP under hypoxic conditions over a 24 h period.

We first analysed the baseline dynamic behaviour of the calibrated cysteine-centred metabolic network under hypoxic conditions, using cysteine availability consistent with the experimental setting. Numerical integration of the model over a 24 h period reveals a consistent sequence of metabolic responses (Figure 2). Intracellular cysteine concentrations decline rapidly during the early phase of the simulation and remain low thereafter, reflecting sustained net consumption within the model structure.

As cysteine availability decreases, GSH levels decline continuously over time, indicating that the antioxidant capacity is constrained by limited precursor supply and that no compensatory mechanism is sufficient to stabilise the GSH concentrations under hypoxia. As a consequence, reactive oxygen species (ROS) accumulate monotonically throughout the simulation window. The concurrent decrease in GSH and increase in ROS indicate a progressive weakening of the redox buffering capacity rather than maintenance of redox homeostasis.

The ROS dynamics do not reach saturation or equilibrium within the simulated time window, suggesting that detoxification processes represented in the model are insufficient to offset hypoxia-associated ROS production. The system therefore evolves away from a redox steady state, displaying sustained oxidative pressure rather than dynamic balance.

Hydrogen sulfide (H_2_S) exhibits a transient increase at early and intermediate times, followed by stabilisation at lower concentrations. Within the model, this behaviour arises from the temporary allocation of cysteine flux toward H_2_S-producing reactions, combined with degradation and declining substrate availability at later times. The cellular energetic state, represented by ATP concentration, shows a gradual and continuous decline over time, reflecting the reduced efficiency of energy-generating processes under hypoxic conditions and the absence of energetic recovery within the simulated timeframe.

**Figure 3:**
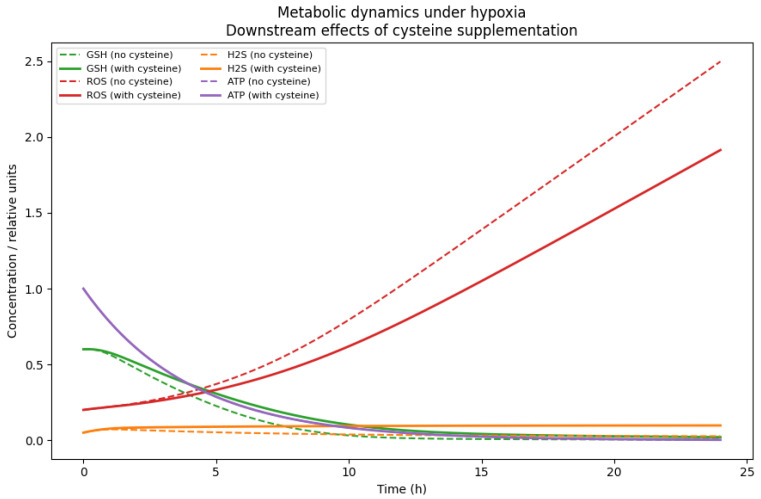
Simulated temporal dynamics of intracellular GSH, ROS, H_2_S, and ATP under hypoxia (H=1) with and without cysteine supplementation. Solid lines denote simulations with cysteine supplementation, whereas dashed lines correspond to cysteine-depleted conditions.

While these simulations characterise the intrinsic hypoxic dynamics of the calibrated network, they do not isolate the specific contribution of cysteine availability from other structural aspects of the model. To explicitly disentangle the causal role of cysteine, we next simulated the system under hypoxic conditions with and without extracellular cysteine supplementation, while keeping all intracellular parameters fixed (Figure 3).

Under cysteine-depleted conditions, the GSH levels decline progressively, resulting in sustained ROS accumulation and a marked reduction in ATP availability. In contrast, cysteine supplementation preserves the intracellular redox buffering capacity by sustaining the GSH levels, limits ROS accumulation, and maintains higher ATP levels throughout the simulation window (Figure 3). Notably, ATP stabilisation under hypoxia emerges from coordinated effects on both antioxidant defence and alternative energy-supporting pathways, including enhanced H_2_S availability. Together, these results demonstrate that cysteine acts as a protective metabolic input under hypoxic stress, rather than merely reflecting condition-dependent parameter shifts.

Although direct intracellular measurements are not used for calibration, the qualitative ordering of simulated intracellular trends is consistent with the experimentally reported directionality in ovarian cancer hypoxia models. In particular, studies have observed the depletion of intracellular GSH and accumulation of oxidative stress markers under cysteine limitation, as well as the partial restoration of redox balance upon cysteine supplementation. The present simulations reproduce these directional patterns (GSH ↓, ROS ↑ under depletion; attenuation under supplementation), thereby providing indirect qualitative validation of the inferred intracellular dynamics. No claim of quantitative intracellular matching is made.

### 6.3. Global Sensitivity Analysis Identifies Key Metabolic Control Points

To quantitatively identify the parameters governing cellular fitness under hypoxia, a variance-based global sensitivity analysis was performed using Monte Carlo sampling over the biologically admissible parameter ranges. The final ATP concentration at 24 h was used as a proxy for cellular fitness.

The resulting first-order Sobol indices are summarised in Table 3.

Small negative values obtained for $k_{\mathrm{ROS}}$ and β ($O(10^{-5})$) arise from numerical estimation noise and are therefore reported as zero.

First-order effects indicate comparable primary influence of $V_{G}$ (maximal cysteine-to-glutathione flux) and $k_{\mathrm{in}}$ (cysteine uptake rate) on ATP-related outputs, with secondary contribution from $V_{S}$ (maximal cysteine-to-H_2_S flux). In contrast, parameters governing basal and hypoxia-amplified ROS production ($k_{\mathrm{ROS}}$ and β) exhibited negligible first-order variance contribution within the explored domain.

Total-order indices confirm that $k_{\mathrm{in}}$ and $V_{G}$ remain the dominant contributors to output variance when parameter interactions are taken into account, while $V_{S}$ exerts a moderate influence, and the ROS-production parameters remain weakly contributory. These sensitivity magnitudes therefore reflect quantitatively relevant control within physiologically constrained parameter ranges, rather than unrestricted structural dominance.

Pairwise bootstrap correlations (Section 4.5) indicate moderate compensatory behaviour between $k_{\mathrm{in}}$ and $V_{G}$, consistent with the shared dependence of intracellular allocation on cysteine availability. Nevertheless, no near-singular dependencies were detected, and the global sensitivity ranking remains stable within the calibrated neighbourhood.

#### Structural Robustness to ATP Formulation

To evaluate the structural robustness of the identified control hierarchy, the simulations were repeated under an alternative ATP formulation in which oxidative phosphorylation efficiency was independent of the glutathione concentration (ATP production proportional only to 1−H). Although the absolute ATP levels differed under this alternative structure, the relative ranking of Sobol indices remained qualitatively preserved, with $k_{\mathrm{in}}$ and $V_{G}$ retaining primary influence. This indicates that the observed control hierarchy is not an artefact of the specific ATP representation adopted but rather a robust property within the imposed reduced cysteine-centred network architecture and bounded parameter domain.

### 6.4. Robustness of the Metabolic Adaptation Regime

Across the explored parameter space, the model displays robust qualitative behaviour, with no abrupt transitions or numerical instabilities.

The sensitivity hierarchy identified in Section 6.3 remains consistent across the admissible parameter domain, with the comparable primary influence of $k_{\mathrm{in}}$ and $V_{G}$ and secondary contribution from $V_{S}$. No parameter exhibited abrupt dominance shifts under moderate parameter variation.

Collectively, these results indicate that hypoxic adaptation in ovarian cancer cells is governed by coordinated cysteine uptake and allocation, rather than by a single isolated flux parameter. The robustness of this hierarchy supports the interpretation that metabolic control emerges from balanced cysteine partitioning within the reduced network architecture.

### 6.5. Regime Exploration Under Hypoxia

To investigate how oxygen availability shapes system-level metabolic behaviour, the hypoxia parameter *H* was systematically varied from normoxia (H=0) to severe hypoxia (H=1). For each value of *H*, steady-state levels of ATP and reactive oxygen species (ROS) were computed from the model.

**Figure 4:**
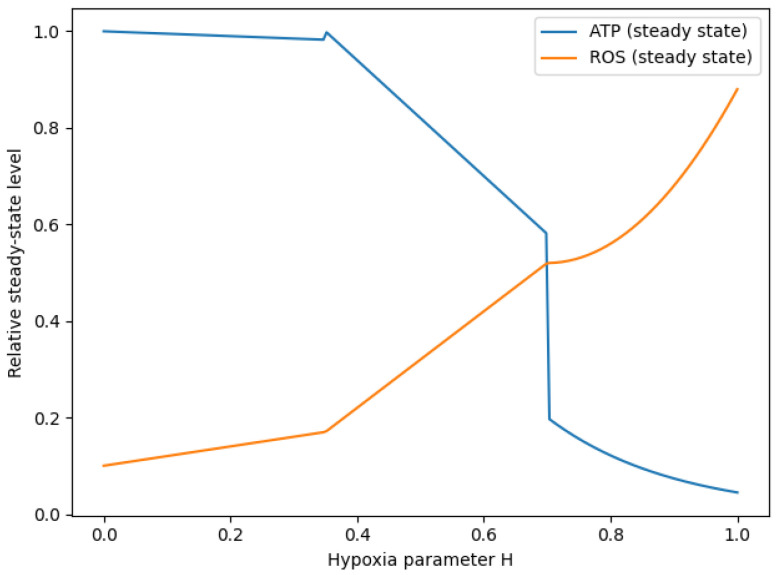
Steady-state ATP and ROS levels as functions of the hypoxia parameter *H*. Parameter continuation identifies three qualitatively distinct response regions: a low-hypoxia region with stable ATP and low ROS, an intermediate region with progressive ATP depletion and ROS accumulation, and a high-hypoxia region characterised by marked energetic impairment and elevated oxidative burden. No formal bifurcation analysis was performed; transitions correspond to sharp but continuous shifts in steady-state values rather than mathematically established bifurcation points.

As shown in Figure 4, this parameter scan reveals the emergence of three operationally distinct metabolic response regions. At low hypoxia, ATP levels remain largely stable while ROS levels are low, indicating a regime of sustained energetic capacity. At intermediate values of *H*, ATP exhibits a monotonic decline accompanied by a gradual accumulation of ROS, consistent with progressive energetic depletion. At high hypoxia, ATP levels collapse, and ROS increase sharply, defining a high oxidative burden regime.

These regions do not correspond to bifurcation points but rather to continuous yet steep variations in steady-state observables as *H* increases.

### 6.6. Linear Stability Analysis and Structural Neutrality

To formally assess whether the hypoxia-dependent transitions observed in Figure 4 correspond to true dynamical bifurcations, a linear stability analysis of the steady state was performed across the full hypoxia domain H∈[0,1].

Let $X={\left(C,G,R,S,A\right)}^{⊤}$ denote the state vector. The system (3)–(7) can be compactly written as $\dot{X}=F\left(X;H\right)$.

For each value of *H*, the system was numerically integrated to convergence, and the Jacobian matrix

$$J\left(X^{*};H\right)=\frac{\partial F}{\partial X}{|}_{X=X^{*}}$$

was evaluated at the computed steady state $X^{*}$. Let $\lambda_{i}\left(H\right)$ denote the eigenvalues of $J(X^{*};H)$. The dominant real part satisfies

(18) $$\max_{H\in [0,1]}ℜ\left(\lambda_{\max}\left(H\right)\right)<10^{-15},$$

indicating the absence of eigenvalues with a strictly positive real part and therefore the exclusion of local bifurcations within the explored physiological range. Representative numerical values across the hypoxia domain remained within floating-point precision of zero.

**Figure 5:**
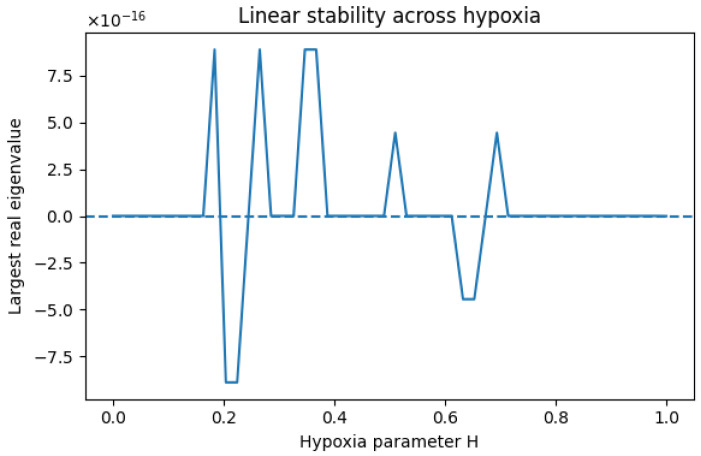
Largest real part of the Jacobian eigenvalues as a function of hypoxia level *H*. Eigenvalues remain non-positive across the entire domain ($|\lambda |<10^{-15}$), confirming absence of bifurcation and preservation of linear stability.

Interestingly, the eigenvalue spectrum consistently contains a structurally zero eigenvalue (Figure 5). This neutral direction arises from the bilinear redox interaction term $k_{R}GR$, which imposes at steady state the constraint

(19) $$k_{R}GR=\left(1+\beta H\right)k_{\mathrm{ROS}},$$

thereby defining a one-dimensional invariant manifold within the (G,R) subsystem. While this induces structural degeneracy in the redox variables, all transverse eigenvalues possess strictly negative real parts.

Consequently, the steady state is normally attractive: perturbations orthogonal to the neutral manifold decay exponentially, and no instability emerges across the hypoxia domain. The hypoxia-dependent regimes described in Section 6.5 therefore correspond to continuous quantitative transitions in steady-state levels rather than bifurcation-driven qualitative state changes.

This neutral direction reflects the minimal redox representation adopted in the reduced network and does not imply multistability or alternative stable states.

This result reinforces that the observed regime structure reflects graded metabolic adaptation within a globally stable reduced network architecture.

## 7. Discussion

This study develops a reduced flux-constrained mathematical framework to analyse cysteine-centred metabolic adaptation under hypoxic conditions in ovarian cancer cells. The model provides a quantitative systems-level explanation for the experimentally observed dependence of ovarian cancer cells on cysteine under hypoxia, a pro-oxidative stress condition [8] that characterises regions of the ovarian cancer microenvironment [9,10].

The model does not claim quantitative prediction of intracellular metabolite concentrations. Rather, intracellular dynamics are inferred as latent variables constrained by extracellular flux consistency and biologically supported qualitative behaviour reported in independent studies [6,7,7]. The framework therefore provides mechanistic plausibility under flux constraints rather than direct intracellular validation.

In ovarian cancer, hypoxia and oxidative stress coexist and are associated with the upregulation of regulatory factors such as hypoxia inducible factor–1α (HIF–1α) and nuclear factor erythroid 2-related factor 2 (NRF2) [11]. These transcriptional programmes contribute to redox adaptation and may promote resistance to conventional chemotherapy [12,13]. Within this biological context, the present model isolates cysteine allocation as a determinant of redox–energetic coupling under experimentally imposed hypoxia.

It is essential to state explicitly the epistemic scope of the framework. Calibration relies exclusively on extracellular metabolite fluxes measured over a 24 h interval. Consequently, intracellular state variables (GSH, ROS, H_2_S, ATP) are not directly validated experimentally within this study. Rather, their trajectories represent dynamically inferred latent variables constrained by (i) extracellular flux consistency, (ii) biochemical plausibility, and (iii) qualitative agreement with the independent literature. The model therefore provides constrained mechanistic plausibility rather than quantitative intracellular validation.

Within experimentally supported parameter ranges, GSH synthesis capacity emerges as the dominant determinant of ATP stability. This observation is consistent with previous experimental studies demonstrating that cysteine-sustained GSH turnover is crucial for ovarian cancer cell adaptation to hypoxia and resistance to carboplatin cytotoxicity [6]. The model does not imply that GSH directly produces ATP; rather, it captures the requirement of adequate redox buffering for sustained oxidative phosphorylation efficiency. Thus, the apparent predominance of GSH synthesis reflects the central role of redox control under pro-oxidative hypoxic stress.

In the clinical setting, cysteine is the predominant thiol in peripheral blood serum and ascitic fluid of cancer patients [6], and both cysteine and homocysteine levels have been proposed as biomarkers distinguishing healthy women, women with benign ovarian tumours, and women with ovarian cancer [6]. The present modelling results therefore provide a mathematical formalisation of experimentally observed cysteine dependency. However, no direct quantitative comparison with patient-level metabolomic ranges is performed here; extrapolation to clinical stratification remains a hypothesis requiring independent validation.

The extracellular cysteine concentrations explored in the simulations lie within the same order of magnitude as values reported in serum and ascitic fluid metabolomic studies, supporting the physiological plausibility of the imposed input ranges. However, no direct quantitative patient-level calibration is performed, and translational inference remains hypothetical pending dedicated clinical validation.

Beyond GSH synthesis, the model quantifies the contribution of cysteine-derived H_2_S. The results underscore the metabolic redundancy and flexibility in cysteine allocation. In the reduced network considered, H_2_S exerts a secondary stabilising influence on ATP levels. This is consistent with the reported roles of H_2_S as both an antioxidant molecule contributing to redox regulation [14] and an electron donor to the mitochondrial electron transport chain [15]. Importantly, in the present framework, H_2_S contribution is represented phenomenologically rather than via explicit modelling of electron transport kinetics.

ATP levels in the simulations remain above critical thresholds in parameter regimes where cysteine allocation toward both GSH and H_2_S is preserved. Within the confines of the reduced network, this supports the hypothesis that cysteine metabolism contributes to ovarian cancer cell fitness under oxidative stress conditions such as hypoxia and drug exposure [16]. However, this conclusion is conditional upon the imposed network architecture and bounded parameter ranges.

A critical methodological point concerns metabolic simplification. The model intentionally excludes glycolysis, fatty acid oxidation, NADPH-producing reactions, and one-carbon metabolism. These pathways undeniably influence ATP production and redox control. Their omission should not be interpreted as biological irrelevance but as a deliberate reduction strategy to avoid underdetermined parameter spaces and uncontrolled compensatory flux redistribution. Consequently, the identified control hierarchy is conditional on this reduced cysteine-centred subnetwork.

Although no bifurcation structure was detected, linear stability analysis confirmed that hypoxia-induced regime transitions correspond to continuous steady-state deformations within a globally stable reduced network.

Finally, the model predicts that the simultaneous disruption of cysteine uptake and downstream allocation pathways may destabilise the redox–energetic balance under oxidative stress. Within the restricted scope of the current framework, this suggests a potential systems-level rationale for targeting cysteine metabolism in combination therapeutic strategies aimed at overcoming hypoxia-associated chemoresistance. Nevertheless, predictive use in therapeutic planning would require integration with intracellular validation, expanded metabolic representation, and patient-derived quantitative data.

## 8. Limitations

While the proposed model captures key features of cysteine-centred metabolic adaptation to hypoxia, its scope and inferential limits must be clearly delineated.

First, the model calibration relies exclusively on extracellular metabolite fluxes derived from exometabolomic measurements over a finite experimental interval. Consequently, the model cannot identify intracellular metabolite pool sizes, transport kinetics, or enzyme-level regulatory parameters from exometabolome data alone. Intracellular states are inferred as dynamically consistent latent variables constrained by extracellular flux balance and biochemical plausibility rather than as independently validated quantitative measurements. Accordingly, the conclusions of the model are restricted to flux-consistent mechanistic inference under controlled in vitro conditions.

Second, exchange processes between extracellular and intracellular compartments are represented implicitly rather than through explicit transport equations. As a result, microenvironmental gradients, transporter saturation effects, and dynamic exchange kinetics are not resolved.

Third, oxygen availability is represented by a single abstract hypoxia parameter *H*, rather than by explicit modelling of oxygen transport, diffusion, or consumption. The framework therefore does not capture spatial or temporal heterogeneity in oxygen tension, nor feedback coupling between mitochondrial respiration and oxygen depletion.

Fourth, although the experimental data originate from large populations of ovarian cancer cells, including two distinct cell lines, the model describes an average intracellular metabolic state. It does not account for cell-to-cell heterogeneity, stochastic variation, proliferation dynamics, or cell death processes.

The metabolic structure itself is intentionally restricted to a minimal cysteine-centred subnetwork. Core pathways known to contribute to hypoxic adaptation, including glycolysis, fatty acid oxidation, NADPH-generating reactions, and the methionine one-carbon cycle, are not explicitly represented. This reductionist architecture was adopted to avoid underdetermined parameter spaces and uncontrolled compensatory flux redistribution. However, it necessarily limits the metabolic completeness of the framework. Incorporation of these pathways in future model extensions will be required to evaluate whether the identified control hierarchy remains stable under broader metabolic coupling conditions.

From an identifiability perspective, explicit incorporation of glycolysis, NADPH-generating reactions, fatty acid oxidation, and one-carbon metabolism would introduce a substantial increase in dimensionality, adding more than 15–20 additional kinetic parameters in minimal representations. Given calibration based solely on extracellular fluxes over a finite time interval, such expansion would lead to severely underdetermined parameter spaces and amplified compensatory flux redistribution. The present reduction strategy therefore reflects a controlled trade-off between the biological scope and inverse-problem well-posedness.

## 9. Conclusions

We present a reduced mechanistic mathematical framework linking cysteine metabolism to hypoxic adaptation in ovarian cancer cells. By calibrating the model exclusively against experimentally derived extracellular metabolite fluxes and constraining the parameter uncertainty within biologically admissible ranges, we establish a flux-consistent systems-level description of cysteine allocation between GSH synthesis and H_2_S production under hypoxic stress.

Within the imposed network structure and calibrated parameter domain, GSH synthesis emerges as the principal determinant of ATP maintenance, while H_2_S production provides a secondary stabilising contribution. These findings should be interpreted as conditional on the reduced cysteine-centred subnetwork considered and do not imply exhaustive metabolic control dominance.

The framework does not uniquely identify intracellular metabolite pool sizes, enzyme-level kinetics, or transport dynamics; rather, it defines dynamically feasible intracellular behaviours consistent with extracellular constraints and established biochemical mechanisms. Its primary contribution lies in formalising experimentally observed cysteine dependency into a mathematically disciplined inference structure.

Future developments should incorporate additional metabolic pathways, including glycolysis, NADPH-generating reactions, fatty acid oxidation, and one-carbon metabolism, as well as explicit oxygen dynamics and population-level effects, in order to evaluate compensatory flux redistribution and broader metabolic coupling. Integration with intracellular measurements and quantitative patient-derived metabolomic data, including peripheral blood serum and ascitic fluid profiling, will be required to assess the predictive validity and translational relevance.

In its present form, the model provides a constrained systems-level platform for hypothesis generation and controlled expansion toward more comprehensive representations of hypoxic cancer metabolism.

## Figures and Tables

**Table 1:** Bootstrap-based parameter estimates and practical identifiability metrics (B=500).

Parameter	Mean	Std	2.5%	97.5%	CV (%)
$k_{\mathrm{in}}$	0.2920	0.0123	0.2658	0.3134	4.21
$V_{G}$	0.1874	0.0150	0.1588	0.2187	7.99
$V_{S}$	0.2918	0.0093	0.2736	0.3000	3.19
$k_{\mathrm{ROS}}$	0.01034	0.00046	0.00968	0.01127	4.43
β	0.50001	0.00005	0.499999	0.500044	0.009

**Table 2:** Comparison between experimentally inferred metabolic fluxes ($v_{exp}$) and mean fluxes predicted by the mathematical model (${\bar{v}}_{model}$) under hypoxic conditions (ES2 cell line).

Metabolite	$M_{\mathrm{initial}}$ (mM)	$M_{\mathrm{final}}$ (mM)	$v_{\exp}$ (mM h^−1^)	${\bar{v}}_{\mathrm{model}}$ (mM h^−1^)	Relative Deviation (%)
Cysteine	0.2626	0.0671	0.0081	0.0076	6.2
Glutamate	0.0103	0.1470	−0.0057	−0.0053	7.0
Glutamine	2.3194	0.7627	0.0652	0.0619	5.1
Methionine	0.1561	0.0970	0.0025	0.0026	3.8
Glucose	20.3972	4.6909	0.6544	0.6210	5.1

**Table 3:** Global sensitivity analysis of model parameters with respect to final ATP levels at 24 h. First-order ($S_{1}$) and total-order ($S_{T}$) Sobol indices quantify primary and interaction effects within the physiologically constrained parameter domain.

Parameter	First-Order ($S_{1}$)	Total-Order ($S_{T}$)
$k_{\mathrm{in}}$	0.3023	0.4485
$V_{G}$	0.3145	0.4353
$V_{S}$	0.1934	0.2807
$k_{\mathrm{ROS}}$	0.0000	0.0000
β	0.0000	0.0000

## Data Availability

The raw data are published in a public repository. http://dx.doi.org/10.21228/M8GV98.
